# Supplementary material for: CARD19 Interacts with Mitochondrial Contact Site and Cristae Organizing System Constituent Proteins and Regulates Cristae Morphology
Source: Cells. 2022 Mar 31;11(7):1175. doi: 10.3390/cells11071175 (PMC8997538; doi:10.3390/cells11071175)
Supplement: Supplementary file 1 [file cells-11-01175-s001.zip › Supplemental Figures.pdf]

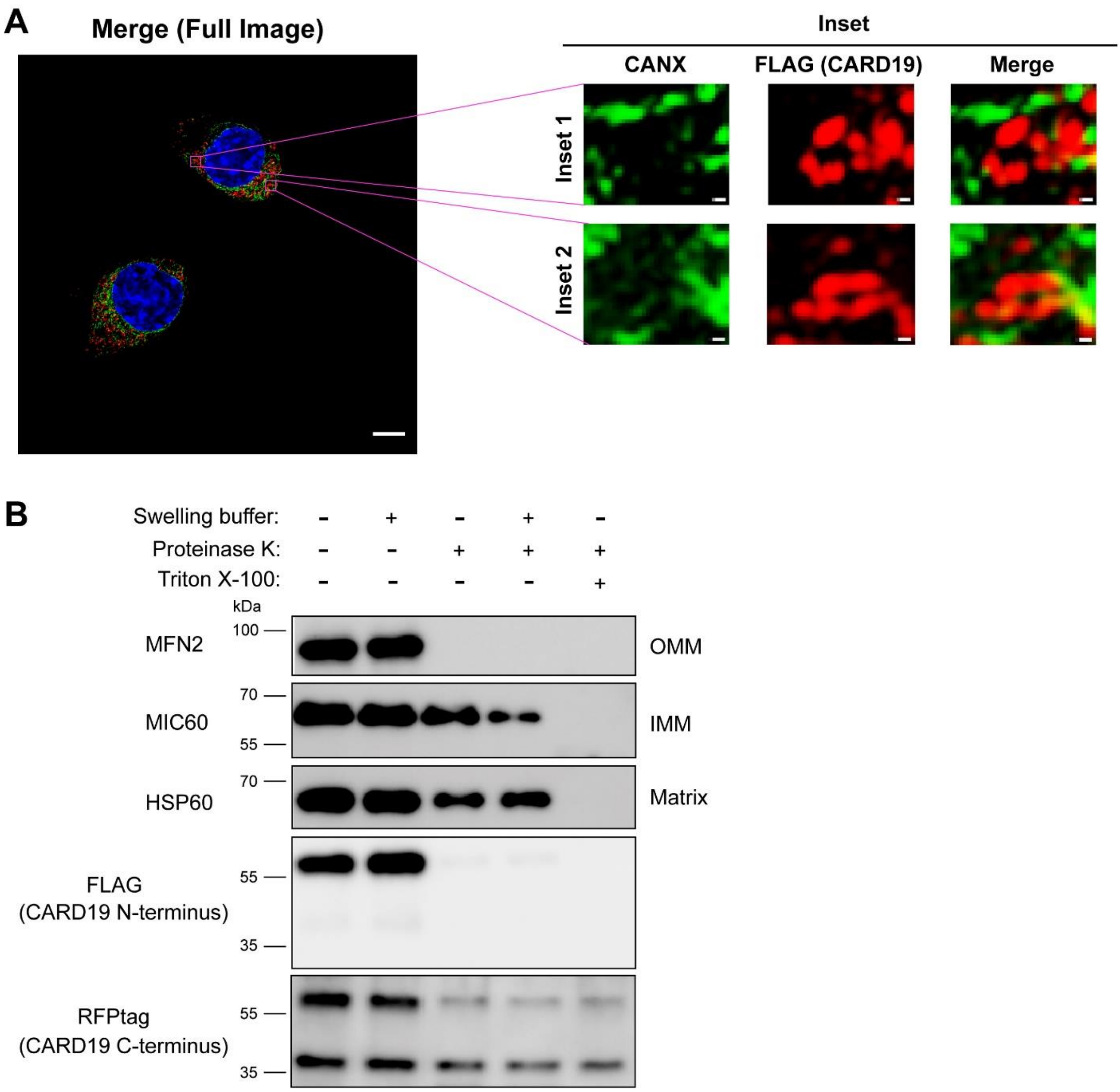

Supplemental Figure S1: CARD19 is an outer mitochondrial membrane protein that may contact the endoplasmic reticulum. (A) *Card19*$^{-/-}$ immortalized macrophages reconstituted with 3xFLAG-CARD19 were labeled with anti-Calnexin (CANX) and anti-FLAG and imaged via SIM. Insets are maximum intensity projections of position subsets within the acquired z-stack (inset 1: positions 13 - 16; inset 2: positions 13 - 16; inset 3: positions 16 - 21). Scale bars in full-sized images correspond to 5 µm. Scale bars in the inset images correspond to 0.1 µm. (B) Mitochondria were isolated from a *Card19*$^{-/-}$ fibroblast cell line stably expressing 3×FLAG-CARD19-TagRFP-T using a Qiagen Q proteome mitochondrial isolation kit and probed for the indicated proteins via western blot. Equal concentrations of mitochondria were left untreated or treated with Proteinase K (50 µg/ml) in the presence or absence of swelling buffer or Triton X-100 (1%) for 20 minutes on ice.

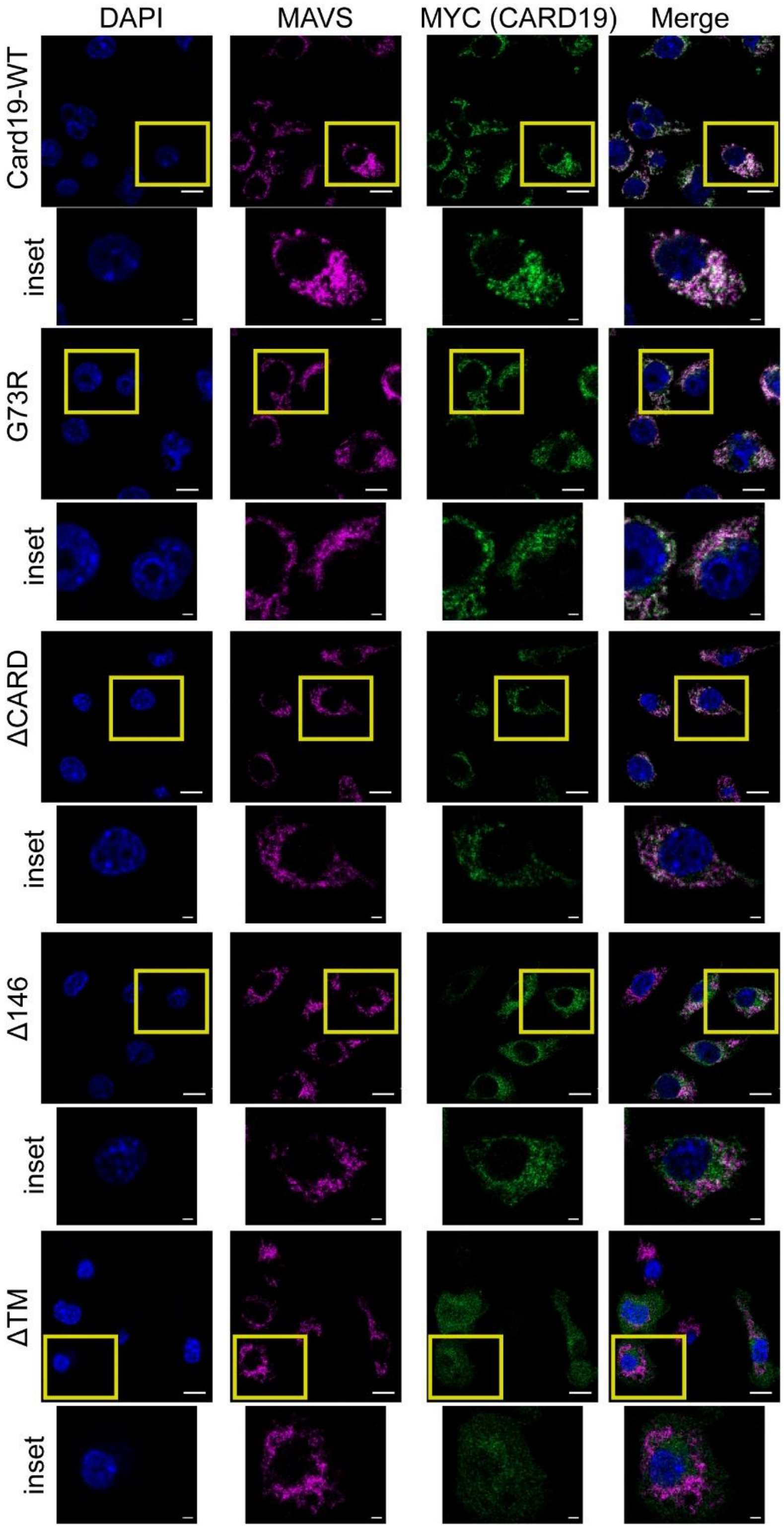

Supplemental Figure S2: Myc-tagged CARD19 and MAVS exhibit similar patterns of mitochondrial distribution. *Card19*$^{-/-}$ immortalized macrophages ectopically expressing MYC-tagged mutants of CARD19 were probed for anti-MYC and anti-MAVS, an outer mitochondrial

membrane (OMM) marker, and imaged via SIM. Insets correspond to yellow boxes in the full images. Scale bars correspond to 2 µm in full images and 0.2 µm in insets.

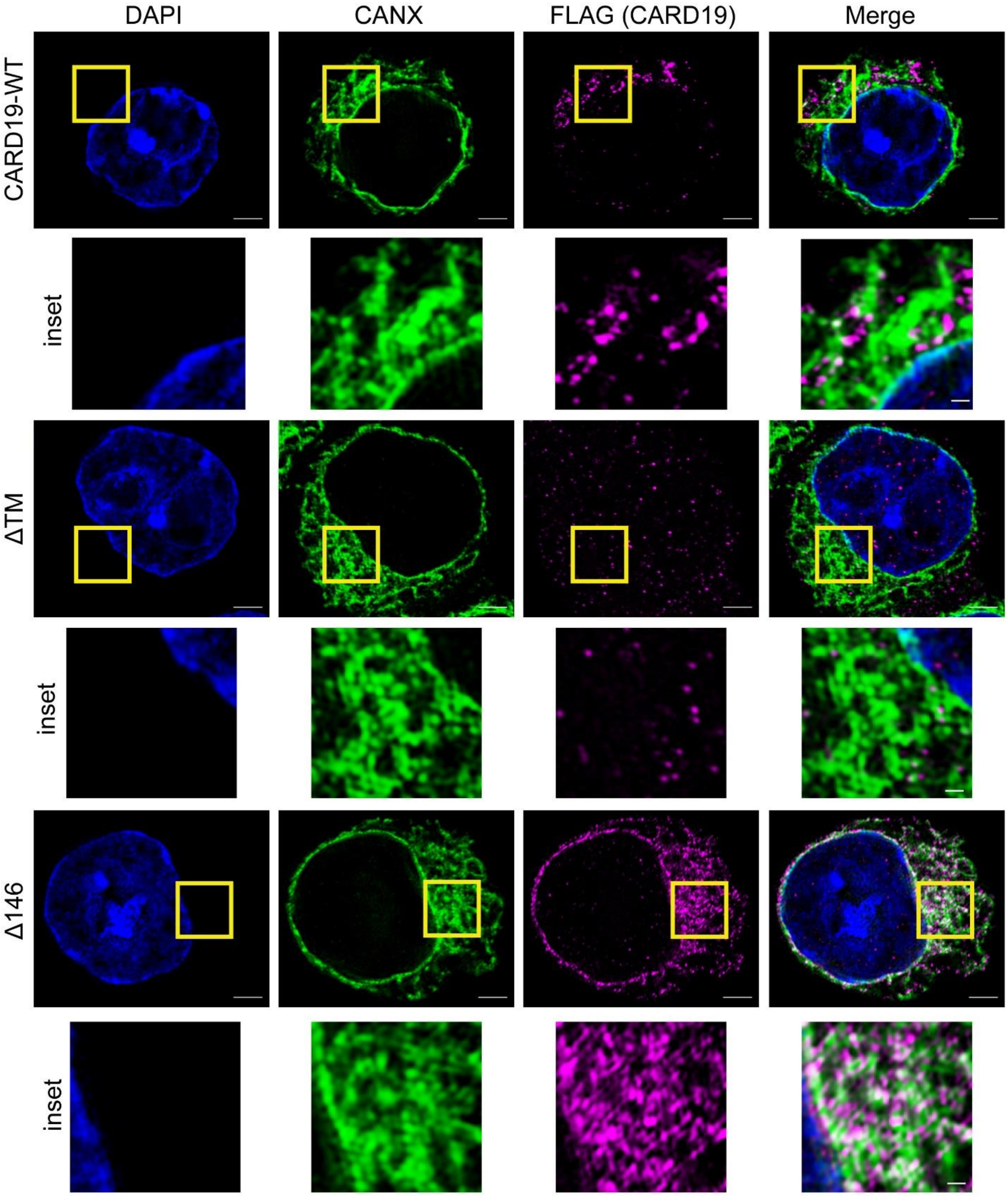

Supplemental Figure S3: The CARD19-Δ146 mutant exhibits a subcellular distribution pattern similar to the ER marker Calnexin. *Card19*$^{-/-}$ immortalized macrophages ectopically expressing FLAG-tagged WT and mutant forms of CARD19 were probed for anti-FLAG and

anti-CANX, an endoplasmic reticulum marker, and imaged via SIM. Insets correspond to yellow boxes in the full images. Scale bars correspond to 5 µm in full images and 0.2 µm in insets.

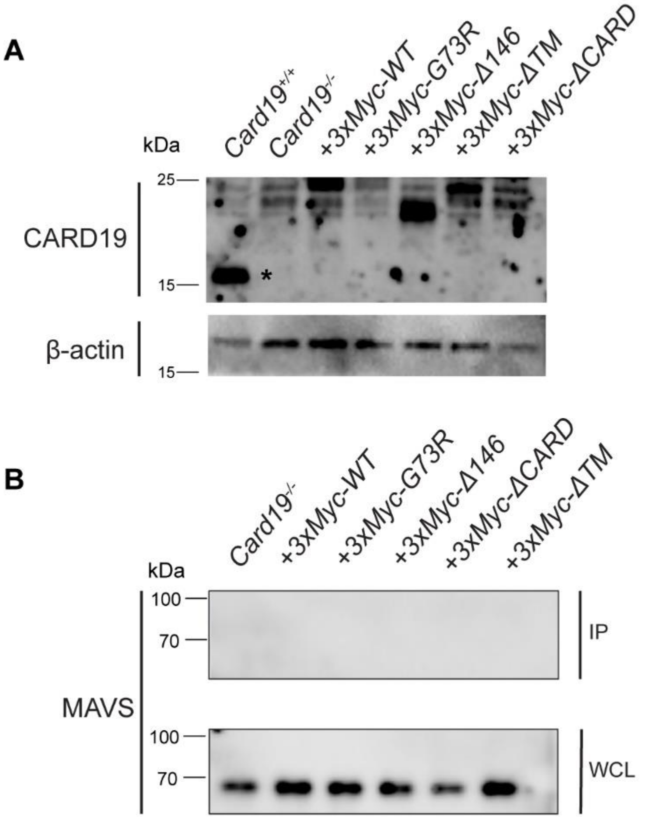

C

Protein	3xMyc-CARD19-FL	3xMyc-CARD19-G73R	3xMyc-CARD19-Δ146	3xMyc-CARD19-ΔTM	*Card19*$^{-/-}$
SAMM50	14	4	2	2	0
MIC19	6	3	2	2	3
MIC60	59	30	14	22	6
MTX2	2	0	0	0	0
MIC25	2	3	0	2	1

Supplemental Figure S4: Mass spectrometry analysis indicates decreased association of CARD19 mutants with MICOS complex proteins. (A) *Card19*$^{-/-}$ immortalized macrophages ectopically expressing 3xMyc-CARD19-FL or derivative mutants were lysed, cleared via centrifugation, immunoprecipitated with ChromoTek Myc affinity or control beads, and analyzed via mass spectrometry. (B) *Card19*$^{-/-}$ immortalized macrophages expressing 3xMyc-CARD19-WT or the indicated mutants were lysed, cleared via centrifugation, pre-cleared with Sepharose G beads, incubated with an anti-Myc antibody, and incubated with Sepharose G beads. Immunoprecipitates and whole cell lysates were then run on 4-20% Tris-glycine gels, transferred to nitrocellulose membrane, and immunoblotted with anti-MAVS. (C) Whole cell lysates of *Card19*$^{+/+}$ and *Card19*$^{-/-}$ immortalized macrophages expressing 3xMyc-CARD19-WT or the

indicated mutants were analyzed via western blotting for endogenous and ectopic CARD19, as well as β-actin. * denotes endogenous, untagged CARD19.

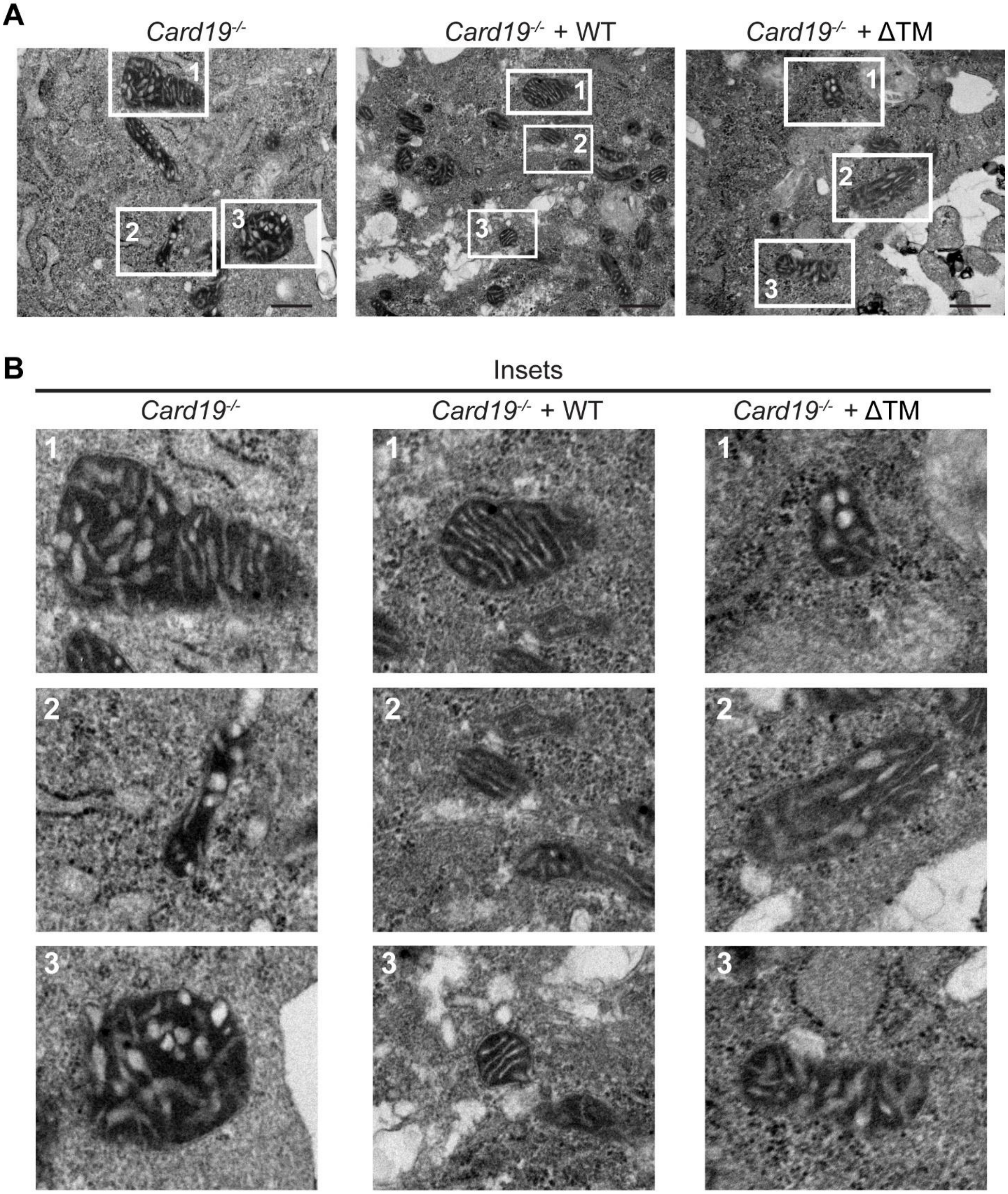

Supplemental Figure S5: ***Card19***$^{-/-}$ **fibroblasts exhibit irregular cristae morphology.** (A) TEM was used to visualize mitochondrial ultrastructure in *Card19*$^{-/-}$ fibroblasts, or *Card19*$^{-/-}$

fibroblasts stably expressing either untagged CARD19-WT or CARD19-ΔTM. *Card19*$^{-/-}$ and *Card19*$^{-/-}$ + CARD19-ΔTM exhibit swollen, vesicular, and irregular cristae. In contrast, *Card19*$^{-/-}$ + CARD19-WT fibroblasts contained mitochondria with a lamellar morphology. Scale bars are 500 nm. (B) Insets of mitochondria corresponding to the white boxed regions in A.
