# Supplementary material for: CARD19 Interacts with Mitochondrial Contact Site and Cristae Organizing System Constituent Proteins and Regulates Cristae Morphology
Source: Cells. 2022 Mar 31;11(7):1175. doi: 10.3390/cells11071175 (PMC8997538; doi:10.3390/cells11071175)
Supplement: Supplementary file 1 [file cells-11-01175-s001.zip › Supplemental Table S1_3x-Myc-Card19_IP-Mass Spec results (significant results only).pdf]

Supplemental Table S1. 3xMyc-CARD19 IP-Mass spec results (significant results only, p<0.05)

protein	accession	Normalized Myc_nanobody (A)	Normalized Resin only (B)	ratio (A/B)	log2 ratio	z score	p-value
Pre-mRNA-processing factor 40 homolog A OS=Mus musculus GN=Prpf40a PE=1 SV=1	Q9R1C7	159.35	1	159.35	7.32	5.61	2.05E-08
DENN domain-containing protein 4C OS=Mus musculus GN=Dennd4c PE=1 SV=1	A6H8H2	107.56	0	117.56	6.88	5.27	1.4E-07
Caspase recruitment domain-containing protein 9 OS=Mus musculus GN=Card9 PE=1 SV=1	A2AIV8	62.74	0	62.74	5.97	4.56	5.16E-06
E3 UFM1-protein ligase 1 OS=Mus musculus GN=Ufl1 PE=1 SV=2	Q8CCJ3	60.75	0	61.75	5.95	4.54	5.61E-06
Centrosomal protein of 170 kDa OS=Mus musculus GN=Cep170 PE=1 SV=2	Q6A065	53.78	0	53.78	5.75	4.38	1.16E-05
Telomere-associated protein RIF1 OS=Mus musculus GN=Rif1 PE=1 SV=2	Q6PR54	50.79	0	51.79	5.69	4.34	1.41E-05
Tyrosine-protein phosphatase non-receptor type substrate 1 OS=Mus musculus GN=Sirpa PE=1 SV=	P97797	51.79	0	51.79	5.69	4.34	1.41E-05
Forkhead box protein P1 OS=Mus musculus GN=Foxp1 PE=1 SV=1	P58462	40.83	0	41.83	5.39	4.10	4.09E-05
Stromal interaction molecule 1 OS=Mus musculus GN=Stim1 PE=1 SV=2	P70302	41.83	0	41.83	5.39	4.10	4.1E-05
Calcium-binding mitochondrial carrier protein Aralar1 OS=Mus musculus GN=Slc25a12 PE=1 SV=1	Q8BH59	80.67	2	40.84	5.35	4.07	4.6E-05
RNA-binding protein 25 OS=Mus musculus GN=Rbm25 PE=1 SV=2	B2RY56	170.30	5	34.26	5.10	3.88	0.000106
Serine/threonine-protein phosphatase PGAM5, mitochondrial OS=Mus musculus GN=Pgam5 PE=1 S	Q8BX10	33.86	1	33.86	5.08	3.86	0.000111
Ral GTPase-activating protein subunit alpha-2 OS=Mus musculus GN=Ralgapa2 PE=1 SV=2	A3KGS3	30.87	0	31.87	4.99	3.80	0.000147
Zinc finger CCCH domain-containing protein 18 OS=Mus musculus GN=Zc3h18 PE=1 SV=1	Q0P678	29.88	1	29.88	4.90	3.72	0.000197
Inosine-5'-monophosphate dehydrogenase 1 OS=Mus musculus GN=Impdh1 PE=1 SV=2	P50096	27.89	0	27.89	4.80	3.65	0.000267
CDK5 regulatory subunit-associated protein 3 OS=Mus musculus GN=Cdk5rap3 PE=1 SV=1	Q99LM2	26.89	0	26.89	4.75	3.60	0.000313
Eukaryotic translation initiation factor 4B OS=Mus musculus GN=Eif4b PE=1 SV=1	Q8BGD9	159.35	6	26.56	4.73	3.59	0.00033
Calcium-binding mitochondrial carrier protein Aralar2 OS=Mus musculus GN=Slc25a13 PE=1 SV=1	Q9QXX4	78.68	3	26.23	4.71	3.58	0.000348
Afadin OS=Mus musculus GN=Mllt4 PE=1 SV=3	Q9QZQ1	25.89	0	25.89	4.69	3.56	0.000368
Caspase recruitment domain-containing protein 19 OS=Mus musculus GN=Card19 PE=1 SV=1; 3xMy	Q9D1I2; MYC(	25.89	0	25.89	4.69	3.56	0.000368
Sorting and assembly machinery component 50 homolog OS=Mus musculus GN=Samm50 PE=1 SV=	Q8BGH2	25.89	0	25.89	4.69	3.56	0.000368
DDRGK domain-containing protein 1 OS=Mus musculus GN=Ddrgk1 PE=1 SV=2	Q80WW9	24.90	0	24.90	4.64	3.52	0.000435
ATP synthase subunit alpha, mitochondrial OS=Mus musculus GN=Atp5a1 PE=1 SV=1	Q03265	162.34	7	23.19	4.54	3.44	0.000586
Brain-specific angiogenesis inhibitor 1-associated protein 2 OS=Mus musculus GN=Baiap2 PE=1 SV=	Q8BKX1	22.91	0	22.92	4.52	3.42	0.000616
MICOS complex subunit Mic19 OS=Mus musculus GN=Chchd3 PE=1 SV=1	Q9CRB9	22.91	0	22.92	4.52	3.42	0.000616
MICOS complex subunit Mic25 OS=Mus musculus GN=Chchd6 PE=1 SV=2	Q91VN4	22.91	1	22.92	4.52	3.42	0.000616
DNA methyltransferase 1-associated protein 1 OS=Mus musculus GN=Dmap1 PE=1 SV=1	Q9JI44	20.91	0	21.91	4.45	3.37	0.00074
Nuclear factor of activated T-cells 5 OS=Mus musculus GN=Nfat5 PE=1 SV=2	Q9WV30	20.91	0	21.91	4.45	3.37	0.00074
Reticulocalbin-2 OS=Mus musculus GN=Rcn2 PE=1 SV=1	Q8BP92	19.92	0	19.92	4.32	3.27	0.001087
Serine/arginine repetitive matrix protein 2 OS=Mus musculus GN=Srrm2 PE=1 SV=3	Q8BTI8	18.92	0	18.92	4.24	3.21	0.001331
Inosine-5'-monophosphate dehydrogenase 2 OS=Mus musculus GN=Impdh2 PE=1 SV=2	P24547	145.41	8	18.18	4.18	3.16	0.001557
Transcription factor 25 OS=Mus musculus GN=Tcf25 PE=1 SV=2	Q8R3L2	17.93	0	17.93	4.16	3.15	0.001642
B-cell differentiation antigen CD72 OS=Mus musculus GN=Cd72 PE=1 SV=2	P21855	16.93	0	16.93	4.08	3.08	0.002042
Leucine zipper protein 1 OS=Mus musculus GN=Luzp1 PE=1 SV=2	Q8R4U7	16.93	0	16.93	4.08	3.08	0.002042
Nuclear receptor corepressor 1 OS=Mus musculus GN=Ncor1 PE=1 SV=1	Q60974	16.93	0	16.93	4.08	3.08	0.002042
Metaxin-2 OS=Mus musculus GN=Mtx2 PE=1 SV=1	O88441	15.93	0	15.93	3.99	3.02	0.002564
MICOS complex subunit Mic60 OS=Mus musculus GN=Immt PE=1 SV=1	Q8CAQ8	170.30	11	15.57	3.96	2.99	0.002791
Histone deacetylase 9 OS=Mus musculus GN=Hdac9 PE=1 SV=2	Q99N13	13.94	0	13.94	3.80	2.87	0.004165
Pre-mRNA-processing factor 40 homolog B OS=Mus musculus GN=Prpf40b PE=1 SV=2	Q80W14	13.94	0	13.94	3.80	2.87	0.004165
DNA annealing helicase and endonuclease ZRANB3 OS=Mus musculus GN=Zranb3 PE=1 SV=1	Q6NZP1	12.95	0	12.95	3.69	2.78	0.005403
GTPase-activating protein and VPS9 domain-containing protein 1 OS=Mus musculus GN=Gapvd1 PE	Q6PAR5	10.96	0	11.96	3.58	2.69	0.007097
NADH-ubiquinone oxidoreductase 75 kDa subunit, mitochondrial OS=Mus musculus GN=Ndufs1 PE=	Q91VD9	10.96	0	11.96	3.58	2.69	0.007097
WAS/WASL-interacting protein family member 1 OS=Mus musculus GN=Wipf1 PE=1 SV=1	Q8K1I7	10.96	1	11.96	3.58	2.69	0.007097
Cyclin-K OS=Mus musculus GN=Ccnk PE=1 SV=3	O88874	9.96	0	9.96	3.32	2.49	0.012894
DNA-directed RNA polymerase III subunit RPC5 OS=Mus musculus GN=Polr3e PE=1 SV=2	Q9CZT4	9.96	0	9.96	3.32	2.49	0.012894
Gasdermin-A OS=Mus musculus GN=Gsdma PE=2 SV=1	Q9EST1	9.96	0	9.96	3.32	2.49	0.012894
M-phase phosphoprotein 9 OS=Mus musculus GN=Mphosph9 PE=2 SV=1	A6H5Y1	9.96	0	9.96	3.32	2.49	0.012894
AP-3 complex subunit beta-1 OS=Mus musculus GN=Ap3b1 PE=1 SV=2	Q9Z1T1	45.81	5	9.16	3.20	2.39	0.016719
Inosine-5'-monophosphate dehydrogenase 1 OS=Mus musculus GN=Impdh1 PE=1 SV=2; Inosine-5'-	P50096; P245	8.96	0	8.96	3.16	2.37	0.01788
LETM1 and EF-hand domain-containing protein 1, mitochondrial OS=Mus musculus GN=Letm1 PE=1	Q9Z2I0	8.96	1	8.96	3.16	2.37	0.01788
Pericentriolar material 1 protein OS=Mus musculus GN=Pcm1 PE=1 SV=2	Q9R0L6	8.96	0	8.96	3.16	2.37	0.01788
Polymerase delta-interacting protein 3 OS=Mus musculus GN=Poldip3 PE=1 SV=1	Q8BG81	8.96	1	8.96	3.16	2.37	0.01788
COBW domain-containing protein 1 OS=Mus musculus GN=Cbwd1 PE=1 SV=1	Q8VEH6	7.97	0	7.97	2.99	2.24	0.025384
Cyclin-dependent kinase 13 OS=Mus musculus GN=Cdk13 PE=1 SV=3	Q69ZA1	7.97	0	7.97	2.99	2.24	0.025384
SH3 domain-containing kinase-binding protein 1 OS=Mus musculus GN=Sh3kbp1 PE=1 SV=1	Q8R550	7.97	0	7.97	2.99	2.24	0.025384
Smith-Magenis syndrome chromosomal region candidate gene 8 protein homolog OS=Mus musculu	Q3UMB5	7.97	0	7.97	2.99	2.24	0.025384
YEATS domain-containing protein 4 OS=Mus musculus GN=Yeats4 PE=2 SV=1	Q9CR11	7.97	0	7.97	2.99	2.24	0.025384
Calcium-binding mitochondrial carrier protein Aralar1 OS=Mus musculus GN=Slc25a12 PE=1 SV=1; (	Q8BH59; Q9Q	21.91	3	7.30	2.87	2.14	0.032547
14-3-3 protein zeta/delta OS=Mus musculus GN=Ywhaz PE=1 SV=1	P63101	6.97	1	6.97	2.80	2.09	0.03705
Coiled-coil and C2 domain-containing protein 1B OS=Mus musculus GN=Cc2d1b PE=1 SV=1	Q8BRN9	6.97	0	6.97	2.80	2.09	0.03705
Complement component 1 Q subcomponent-binding protein, mitochondrial OS=Mus musculus GN=	O35658	6.97	1	6.97	2.80	2.09	0.03705
Cytochrome c oxidase subunit 2 OS=Mus musculus GN=Mtco2 PE=1 SV=1	P00405	6.97	0	6.97	2.80	2.09	0.03705
Endoplasmic reticulum resident protein 29 OS=Mus musculus GN=Erp29 PE=1 SV=2	P57759	6.97	0	6.97	2.80	2.09	0.03705
Homeobox protein cut-like 1 OS=Mus musculus GN=Cux1 PE=1 SV=3	P53564	6.97	0	6.97	2.80	2.09	0.03705
Lamina-associated polypeptide 2, isoforms beta/delta/epsilon/gamma OS=Mus musculus GN=Tmpc	Q61029	6.97	1	6.97	2.80	2.09	0.03705
Methylthioribulose-1-phosphate dehydratase OS=Mus musculus GN=Apip PE=1 SV=1	Q9WVQ5	6.97	1	6.97	2.80	2.09	0.03705
Proline-rich protein 11 OS=Mus musculus GN=Prr11 PE=2 SV=1	Q8BHE0	6.97	0	6.97	2.80	2.09	0.03705
Lamina-associated polypeptide 2, isoforms beta/delta/epsilon/gamma OS=Mus musculus GN=Tmpc	Q61029; Q61(	12.95	2	6.47	2.69	2.00	0.045306

Blue highlight identifies MICOS/MIB proteins identified in this screen

Yellow highlight identifies peptides from 3xMyc-CARD19 (the bait protein immunoprecipitated with the Myc nanobody
